# Short-Circuit Performance Analysis of Commercial 1.7 kV SiC MOSFETs Under Varying Electrical Stress

**DOI:** 10.3390/mi16010102

**Published:** 2025-01-16

**Authors:** Shahid Makhdoom, Na Ren, Ce Wang, Yiding Wu, Hongyi Xu, Jiakun Wang, Kuang Sheng

**Affiliations:** 1 College of Electrical Engineering, Zhejiang University, Hangzhou 310027, China; shahidmakhdoom@zju.edu.cn (S.M.); wangce@zju.edu.cn (C.W.); 22210029@zju.edu.cn (Y.W.); shengk@zju.edu.cn (K.S.); 2 ZJU-Hangzhou Global Scientific and Technological Innovation Center, Hangzhou 311200, China; xuhongyi@zju.edu.cn; 3 Hangzhou Silicon Magic Semiconductor Technology Co., Ltd., Hangzhou 310052, China; jk.wang@silicon-magic.com

**Keywords:** failure mechanisms, short-circuit (SC) robustness, silicon carbide (SiC) MOSFETs, reliability, 1.7 kV SiC MOSFET, 1.2 kV SiC MOSFET

## Abstract

The short-circuit (SC) robustness of SiC MOSFETs is critical for high-power applications, yet 1.2 kV devices often struggle to meet the industry-standard SC withstand time (SCWT) under practical operating conditions. Despite growing interest in higher voltage classes, no prior study has systematically evaluated the SC performance of 1.7 kV SiC MOSFETs. This study provides the first comprehensive evaluation of commercially available 1.7 kV SiC MOSFETs, analyzing their SC performance under varying electrical stress conditions. Results indicate a clear trade-off between SC withstand time (SCWT) and drain-source voltage (V_DS_), with SCWT decreasing from 32 µs at 400 V to 4 µs at 1100 V. Under 600 V, a condition representative of practical use cases in many high-voltage applications, the devices achieved an SCWT of 12 µs, exceeding the industry-standard 10 µs benchmark—a threshold often unmet by 1.2 kV devices under similar conditions. Failure analysis revealed gate dielectric breakdown as the dominant failure mode at V_DS_ ≤ 600 V, while thermal runaway was observed at higher voltages (V_DS_ = 800 V and 1100 V). These findings underscore the critical importance of robust gate drive designs and effective thermal management. By surpassing the shortcomings of lower voltage classes, 1.7 kV SiC MOSFETs can be a more reliable, and efficient choice for operating at higher voltages in next-generation power systems.

## 1. Introduction

Silicon carbide (SiC) MOSFETs have gained significant attention in high-power applications due to their superior thermal stability, faster switching speed, and higher breakdown voltage compared to traditional silicon-based devices [1,2]. The increasing demand for high-voltage and high-efficiency systems in electric vehicles (EVs), renewable energy, and grid applications has accelerated the development of SiC metal-oxide-semiconductor field-effect transistors (MOSFETs) [3,4,5,6]. However, short-circuit (SC) robustness remains a critical reliability challenge, particularly in high-voltage applications [7,8,9].

Despite their extensive study and adoption, 1.2 kV SiC MOSFETs face challenges in achieving reliable short-circuit (SC) robustness under high-voltage conditions, often failing to meet the industry-standard short-circuit withstand time (SCWT) in demanding applications. The emerging 1.7 kV class presents new opportunities for applications requiring higher voltage operation including DC–DC converters, traction, and grid-tied systems, where the effective management of SC performance and thermal stress is crucial [10,11].

Although studies on 650 V, 900 V, and 1200 V SiC MOSFETs demonstrate their ability to sustain higher power densities during SC events [12,13,14], these devices—particularly 1200 V SiC MOSFETs—face challenges in maintaining SCWT beyond a 10 µs SCWT under half-rated drain-source voltage and full-rated gate voltage [15,16,17]. Furthermore, their performance degrades under repetitive SC events [18,19], highlighting unique challenges in designing effective protection circuits and enhancing device reliability [20,21].

The 1.7 kV class of SiC MOSFETs represents a significant advancement in voltage capability, material properties, and structural optimizations. Despite their growing adoption, comprehensive studies evaluating the SC robustness of the emerging 1.7 kV class SiC MOSFETs are notably lacking. In this medium voltage range of SiC MOSFETs, few studies, such as those by Lee et al., have focused on the design and electrical properties of trench structures, highlighting their effectiveness in reducing on-resistance and improving breakdown voltage characteristics [22]. Similarly, Bolotnikov et al., provided an extensive optimization study for 1.7 kV SiC MOSFETs, evaluating trade-offs between short-circuit withstand time (SCWT) and conduction losses through design improvements like reduced source doping and channel width adjustments [23]. These works primarily focus on fabricated prototypes rather than commercially available devices. As a result, the SC performance and failure mechanisms of 1.7 kV SiC MOSFETs under varying electrical stress conditions remain unexplored.

To address these research gaps, this study conducts the first comprehensive evaluation of 1.7 kV SiC MOSFETs, specifically investigating SC performance metrics, and failure mechanisms under varying drain-source voltage (V_DS_: 400 V, 600 V, 800 V, and 1100 V) and gate-source voltage (V_GS_: −5/15 V, −5/18 V, and −5/20 V) conditions. By addressing this research gap, the findings will offer critical insights into the performance and reliability of 1.7 kV SiC MOSFETs, enabling their effective deployment in high-power applications.

This paper is organized as follows: Section 2 details the experimental setup and device characteristics. Section 3 explores the static characteristics, including transfer characteristics, output characteristics, gate-drain capacitance (C_gd_), and breakdown voltage. Section 4 focuses on the short-circuit (SC) analysis under varying drain-source voltage (V_DS_) and gate-source voltage (V_GS_) conditions and a comparison of SC performance metrics, and is supplemented with optical microscopy images of post-SC failure. Finally, Section 5 concludes with a summary of findings and suggestions for future research.

## 2. SC Test Platform and Device Characteristics

Most high-power systems in real world applications run in the 400 V to 1100 V range. Consequently, it is essential to evaluate SC robustness at these practical voltages in order to fully understand device reliability under real world conditions of operation.

To address this need, our study systematically tested 1700 V SiC MOSFETs at voltages ranging from 400 V to 1100 V, covering both moderate and high electrical stress scenarios. Testing up to 1100 V, approximately 2/3 of the device’s rated voltage, aligns with industry practices and reflects realistic operating conditions for high-power systems. Lower voltage tests (400 V and 600 V) offer insights into SCWT under moderate stress, while higher voltage tests (800 V–1100 V) simulate more demanding scenarios, enabling a comprehensive evaluation of device robustness.

We have examined six identical devices with the same specifications, all from the same manufacturer, CREE, under short-circuit (SC) conditions. The key parameters of these devices are listed in Table 1. Testing was conducted under varying DC bus voltages (V_DS_) of 400 V, 600 V, 800 V, and 1100 V, with gate-source voltages (V_GS_) of [−5/20 V] as specified in the datasheet [24]. Additionally, the influence of gate-source voltages was analyzed by testing the devices under varying V_GS_ values (15 V, 18 V, 20 V) at a constant V_DS_ of 600 V.

The short-circuit pulse width is systematically increased by 1 μs until the device fails in all conducted SC tests. In this study, we have analyzed SC waveforms, including V_GS_, I_DS_, and V_DS_, to determine the SCWT in a real-time environment. SCWT is defined as the time a device can sustain without catastrophic failure. Specially, we utilized a sudden and significant drop in V_GS_ as the key indicator for gate degradation initiation. This approach aligns with methodologies commonly employed in the literature, where waveform characteristics provide robust insights into device performance. Moreover, to ensure the reliability of our measurements, after each test, the device was allowed to cool down before initiating the next test to avoid heat buildup within the device.

**Figure 1:**
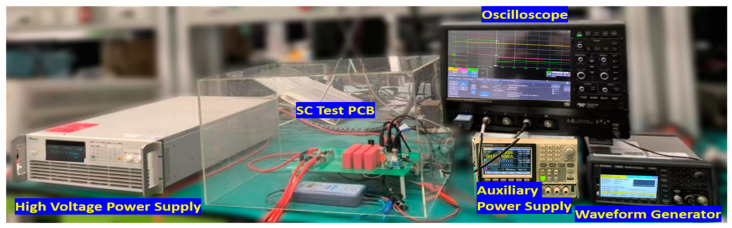
A photograph of the short-circuit characterization test setup is shown. It features the high-voltage power supply, which charges the bus capacitor on the Printed Circuit Board (PCB) and ensures a stable DC bus voltage for the tests. The gate trigger signal, crucial for switching the SiC MOSFETs on and off, is provided by the waveform generator. Simultaneously, the oscilloscope captures key measurements, including the drain-source voltage (V_DS_), drain current (I_DS_), and gate-source voltage (V_GS_) of the SiC MOSFETs during short-circuit testing.

The test platform (Figure 1) consists of a high-voltage, auxiliary power supply, a waveform generator, and an oscilloscope to monitor critical parameters (V_DS_, I_DS_, and V_GS_) during SC events. The SC test PCB integrates a solid-state circuit breaker with an IGBT [25], capacitors (C1, C2, and C3), and a SiC MOSFET driver to control the device under test (DUT). A current shunt was employed to measure the drain current (I_DS_) during the SC event. Further details of the SC test schematic and platform are available in our previous work [26,27].

To ensure the reliability of results, two distinct sets of devices were used. The first set, labeled S1 through S5, was exclusively used for static characterization tests, including transfer characteristics, output characteristics, and C_gd_. These non-destructive tests provided baseline data for static device performance. A single device, S1, was specifically used for a destructive breakdown voltage test to assess the limit of the CREE device’s ability to sustain breakdown voltages. To evaluate the short-circuit test, a second set of devices, SC1–SC6, was dedicated. The SC tests were performed under varying electrical stress in terms of V_DS_ and V_GS_. The devices used in static characterization were not employed in the SC test in order to avoid any pre-stress influence on device performance.

## 3. Static Characteristics Analysis

### 3.1. Output Characteristics

**Figure 2:**
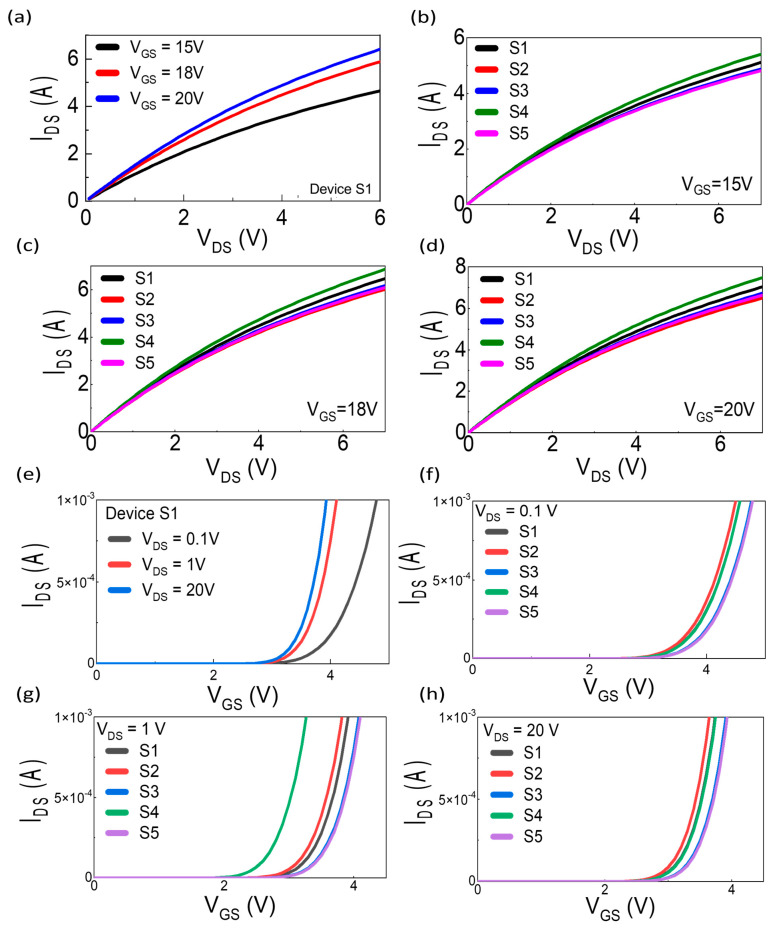
(**a**) Output characteristics of Device S1 at various gate-source voltages (V_GS_ = 15 V, 18 V, and 20 V). (**b**–**d**) Output characteristics of devices S1–S5 at V_GS_ = 15 V, 18 V, and 20 V, respectively, showing trends in I_DS_ versus V_DS_ for multiple devices. (**e**) Transfer characteristics of Device S1 at V_DS_ = 0.1 V, 1 V, and 20 V, illustrating the evolution of I_DS_ with V_GS_ within the same device, with Vth approximately 3.9 V for V_DS_ = 0.1 V. (**f**–**h**) Transfer characteristics of multiple devices (S1–S5) at V_DS_ = 0.1, 1 V, and 20 V, showing consistent I_DS_−V_GS_ trends and minor variations in Vth (ranging from 3.9 V to 4.3 V).

The output characteristic curves of 1.7 kV devices tested under various gate-source voltages are shown in Figure 2a–d.

These curves are obtained by plotting the drain current (I_DS_) as a function of the drain-source voltage (V_DS_). The typical MOSFET behavior can be seen in Figure 2a, where I_DS_ rises initially with increasing V_DS_ within the ohmic region until it reaches a point of saturation.

Figure 2a illustrates the output characteristics of a single device (S1) tested under different gate-source voltage V_GS_ conditions (15 V, 18 V, and 20 V). At V_GS_ = 15 V, the device achieves a saturation current of approximately 5 A when V_DS_ is around 6 V. With V_GS_ increased to 18 V and 20 V, the saturation current rises to around 6 A and slightly higher, respectively, indicating improved current handling with higher gate drive. The devices have shown improved channel conductivity and reduced on-resistance R_ON_ reflecting the higher current with each increasing V_GS_ value (15 V, 18 V, and 20 V).

Figure 2b–d show the comparison of the output characteristics of five devices (S1–S5) from the same vendor under different V_GS_. When V_GS_ = 15 V, the saturation current at V_DS_ = 6 V varied slightly across devices, attributed to device uniformity variations in the value of channel mobility, threshold voltages, and manufacturing tolerance. The device S4 has performed best at this V_GS_ value, and among all tested devices, demonstrated the highest I_DS_ across the V_DS_ range. In Figure 2c, at V_GS_ = 18 V, all devices show an increase in I_DS_, confirming the improved channel conductivity at higher gate voltages. Device S4 again exhibits the highest drain current, while Device S2 shows comparatively lower performance.

In Figure 2d, at V_GS_ = 20 V, the drain current further increases for all devices, reaching their maximum current-handling capability. Device S4 continues to outperform the other devices, while Device S2 exhibits the lowest current values at higher gate voltages, possibly due to slight differences in manufacturing or material quality during fabrication process. The slight variations in measured values among devices fall within manufacturing tolerance. The higher V_GS_ results at higher I_DS_ depict a clear correlation in the output characteristics and indicate superior current handling capabilities of the tested 1.7 kV SiC MOSFETs.

### 3.2. Transfer Characteristics

The transfer characteristic curves of CREE devices were measured at varying V_GS_ values to understand the behavior of I_DS_ with respect to V_GS_ under different V_DS_ conditions. Figure 2e shows the progression of device behavior for a single representative device, S1, tested under three V_DS_ states (V_DS_ = 0.1 V, 1 V, and 20 V), illustrating the evolution of I_DS_ with V_GS_ within the same device. At V_DS_ = 0.1 V, the transfer curves exhibit minimal drain current, which steadily increases at V_DS_ = 1 V, and eventually saturates at V_DS_ = 20 V. This demonstrates the device’s behavior transitioning from the sub-threshold region to the saturation region. This progression highlights the dependence of I_DS_ on both V_GS_ and V_DS_, reflecting the expected behavior for SiC MOSFETs.

For V_DS_ = 0.1 V, Vth is approximately 3.9 V, at which I_DS_ begins to rise significantly (defined as I_DS_ = 1 mA). Similar observations are made for V_DS_ = 1 V and V_DS_ = 20 V, with Vth shifting slightly due to channel modulation effects.

In Figure 2f, at V_DS_ = 0.1 V, all devices (S1–S5) exhibit minimal drain current (I_DS_) at low V_GS_, consistent with sub-threshold operation where the device is not yet fully turned on. As V_GS_ approaches the threshold voltage (Vth), I_DS_ begins to rise significantly, reaching the defined threshold current (I_DS_ = 1 mA). Small variations in I_DS_ across different devices (S1 to S5) can be attributed to sample-to-sample manufacturing differences. Notably, S4 exhibits a slightly lower Vth, around 3.2 V, compared to the typical range of 3.9 V to 4.3 V observed in the other devices, which is within expected manufacturing tolerances.

In Figure 2g, at V_DS_ = 1 V, as V_GS_ increases, the drain current begins to rise more significantly, indicating the device’s transition into the linear or active region. A notable increase in I_DS_ is observed beyond the threshold voltage (Vth). Despite small deviations, the performance of all device samples remains consistent.

In Figure 2h, when V_DS_ = 20 V, under a high drain-source voltage, the devices enter saturation mode as V_GS_ increases beyond Vth, with I_DS_ rising sharply. The saturation mode is a region where I_DS_ become less dependent on V_DS_, and more strongly influenced by V_GS_. All device samples (S1 to S5) display similar trends, demonstrating a high level of uniformity in their behavior.

Across multiple devices (S1–S5), Vth ranges from approximately 3.9 V to 4.3 V (based on the definition of I_DS_ = 1 mA), as observed in Figure 2f–h. The slight variations in measured values among devices fall within manufacturing tolerance.

### 3.3. Gate-Drain Capacitance (C_gd_) Characteristics

**Figure 3:**
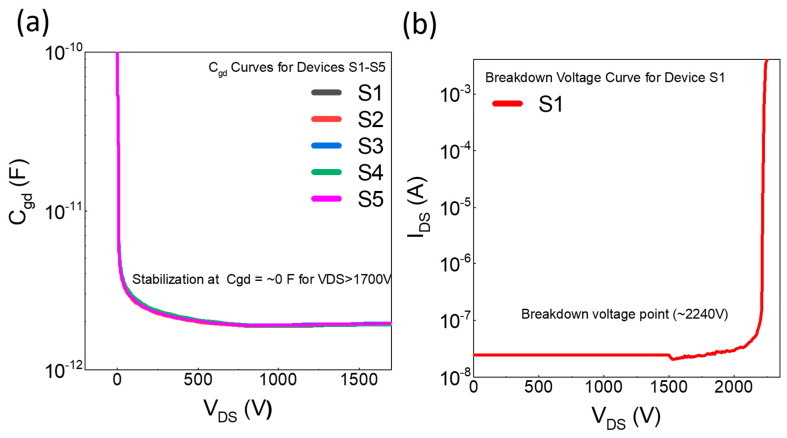
(**a**) The gate-drain capacitance (C_gd_) characteristics of the five devices (S1–S5) measured at a test frequency of 100 kHz and a temperature of 25 °C. The plot reveals a sharp reduction in C_gd_ with increasing V_DS_, leveling off to an almost constant low value at higher voltages. (**b**) Breakdown voltage characteristics of device S1 were measured, highlighting the relationship between I_DS_ and V_DS_, and indicating 2240 V as a breakdown voltage point, at which drain current rises rapidly.

The C_gd_ characteristics of the 1.7 kV SiC MOSFET were evaluated for five devices, labeled S1 through S5, as shown in Figure 3a, tested under 100 kHz frequency at room temperature. The C_gd_ curve reveals a sharp decrease in the C_gd_ with increasing V_DS_ leveling off to an almost constant low value as V_DS_ approaches 1700 V. All five samples show almost no variation in C_gd_ behavior, indicating consistent performance. The rapid drop in C_gd_ with increasing V_DS_ is characteristic of high-voltage SiC MOSFETs, where lower gate-drain capacitance at higher V_DS_ enhances switching efficiency. This stability of C_gd_ at higher V_DS_ values minimizes switching losses, a beneficial property in high-speed switching applications.

### 3.4. Breakdown Voltage Characteristics

The breakdown voltage characteristic was measured for a single device of the 1.7 kV SiC MOSFET, as shown in Figure 3b. This curve plots I_DS_ against V_DS_ to identify the point at which breakdown occurs. The device remains stable with negligible drain current up to approximately 2200 V. A sharp rise in I_DS_ is observed around 2240 V, indicating the onset of breakdown, which is above the nominal 1700 V rating. The high breakdown voltage confirms the robustness of the SiC MOSFET under high-voltage conditions.

## 4. SC Capability

### 4.1. Influence of V_DS_

**Figure 4:**
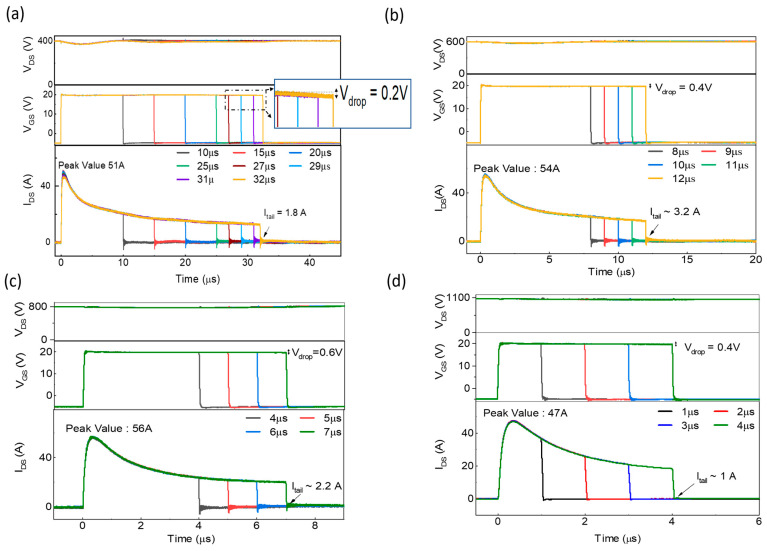
Drain-source voltage (V_DS_), gate-voltage (V_GS_) and SC current (I_DS_) waveforms of devices SC1–SC4, tested under various V_DS_, during normal operation, before failure: (**a**) Device SC1 tested under 400 V, with V_GS_ drop highlighted in the inset; (**b**) Device SC2 tested under 600 V, (**c**) Device SC3 tested under 800 V, and (**d**) Device SC4 tested under 1100 V.

The 1.7 kV SiC MOSFET, SC1, was tested at 400 V with the typical V_GS_ −5/20 V value as specified in the datasheet. The SC waveforms of device SC1, including V_DS_, V_GS,_ and I_DS_, are presented in Figure 4a during normal operation before failure. The peak SC current (I_SC, peak_) value reached 51 A after 0.4 µs.

In the SC process, high local temperatures and the intensive electric field applied to the gate oxide cause higher leakage currents in the gate. Consequently, the V_GS_ of devices decreases gradually as the duration of the SC pulse (T_SC_) increases. As the SC pulse width extended to 32 μs, a tail current (I_tail_) of approximately 1.8 A began to appear, accompanied by a minor reduction in V_GS_ of 0.2 V. This small V_GS_ drop indicates the robustness of the gate drive circuit, which maintains high stability throughout operation. It is noteworthy that this reduction in V_GS_ does not begin immediately after the short circuit starts but becomes more pronounced as T_SC_ progresses, consistent with known gate degradation mechanisms. The inset in Figure 4a highlights this behavior, showing a V_GS_ drop of 0.2 V during the final pulse before failure.

Additionally, V_DS_ remains constant at 400 V throughout the SC event, with no significant voltage drop observed. This indicates robust performance during extended SC exposure prior to failure. The SCWT of SC1 was measured as 32 µs at V_DS_ = 400 V. The minimal tail current and stable gate performance further emphasize the device’s thermal and electrical robustness.

The short-circuit behavior of the SC2 device was evaluated at V_DS_ = 600 V with V_GS_ = −5/20 V. The waveforms for V_DS_, V_GS_, and I_DS_ are shown in Figure 4b. During the SC event, V_DS_ remains stable at 600 V with no significant fluctuations, demonstrating the device’s ability to maintain voltage integrity. A slight V_drop_ of 0.4 V was observed, which is larger than the 0.2 V drop at 400 V, indicating increased stress on the gate drive circuit. However, the drop remains within acceptable limits.

The I_SC, peak_ current reached 54 A and then gradually declined. The tail current (I_tail_) stabilized at 2.7 A before failure. The SCWT was recorded as 12 µs, reflecting a significant reduction compared to 32 µs at 400 V. The shorter SCWT highlights the increased stress on the device under higher V_DS_ conditions, caused by severe power dissipation [28].

The short-circuit behavior of the SC3 device was evaluated at V_DS_ = 800 V with V_GS_ = −5/20 V. The waveforms for V_DS_, V_GS_, and I_DS_ during normal operation are presented in Figure 4c. During the SCWT period, V_DS_ remains stable at 800 V with no significant fluctuations, indicating robust voltage control under increased stress. A significant gate voltage drop of 0.6 V was observed, larger than the drops recorded at 400 V (0.2 V) and 600 V (0.4 V). This suggests higher gate stress and potential gate charge leakage under elevated V_DS_ [29,30]. The I_SC, peak_ of device SC3 reached 56 A, then gradually declined, with an I_tail_ stabilizing at approximately 2.2 A before device failure. The SCWT was recorded as 7 µs, representing a 41% reduction compared to the 600 V test and a 78% reduction compared to the 400 V test.

The SC4 device was tested at V_DS_ = 1100 V with V_GS_ = −5/20 V. The short-circuit waveforms, including V_DS_, V_GS_, and I_DS_, are depicted in Figure 4d. During the short-circuit event, V_DS_ remains stable at 1100 V with no significant fluctuations, demonstrating the device’s robust voltage control under extreme stress. A gate voltage drop of 0.4 V was observed, similar to the 600 V test, reflecting moderate gate leakage under high electric field stress.

The I_SC, peak_ reached 47 A, lower than the 56 A observed at 800 V. This reduction may be attributed to the higher electric field stress limiting the initial current surge. Following the peak, I_DS_ gradually declined, with the I_tail_ stabilizing at approximately 1 A before failure. The SCWT was recorded as 4 µs, the shortest among all tested voltages, indicating the device’s reduced tolerance to high V_DS_ levels.

**Figure 5:**
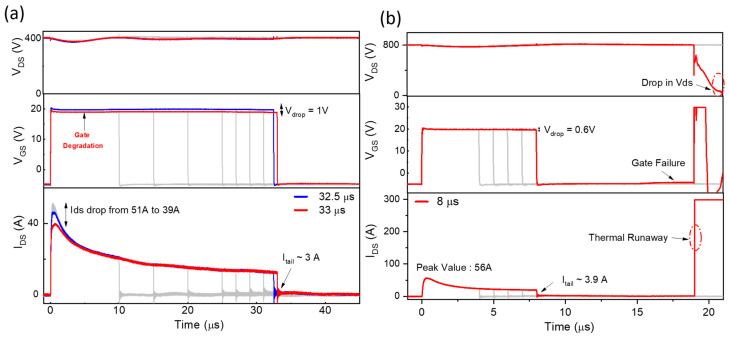
Short-circuit (SC) waveforms, including drain-source voltage (V_DS_), gate voltage (V_GS_), and SC current (I_DS_), are presented for the following: (**a**) Device SC1, tested at 400 V, and (**b**) Device SC3, tested at 800 V. [Gray: normal device operation; blue: onset of device degradation; red: device failure due to gate breakdown or thermal runaway].

Figure 5 illustrates the progression of failure in Device SC1 and Device SC3 under 400 V and 800 V stress, respectively. The SC1 device demonstrated gate degradation as a failure mode under a stress condition of 400 V as shown in Figure 5a. This is evidenced by the gradual decline in I_DS_ and an incremental drop in V_GS_, indicative of stress-induced damage to the gate oxide [31,32]. At SC pulse width (Tsc) = 32.5 µs, the initiation of gate degradation becomes apparent, marked by a small but noticeable decline in V_GS_, accompanied by a reduction in I_DS_. This simultaneous behavior reflects the onset of stress-induced damage to the gate dielectric and its impact on the device’s current conduction capability.

At 33 µs, a sudden and significant V_GS_ drop (1 V) and an increase in tail current to 3 A was observed, suggesting that the applied stress has reached a critical threshold, leading to dielectric breakdown. The reduction in I_DS_ from 51 A to 39 A also supports this observation, suggesting gate degradation leading to device failure. Moreover, upon inspecting the device terminals after the SC test, only gate-source terminals were found to be shorted, with a significant reduction in resistance value (R_GS_ = 290 Ω). In contrast, the drain-source (R_DS_) junction remained in a blocking state, hence V_DS_ was maintained through the SC duration. These electrical changes reflect increased stress on the gate dielectric and the progressive weakening of the device, ultimately leading to gate-dielectric breakdown [29].

Importantly, while degradation begins at 32.5 µs, the SCWT is recorded at 32 µs, as this is the last point before significant degradation occurs. Similar V_GS_ and I_DS_ behavior has also been observed in other SiC MOSFETs under SC conditions [29,33].

These findings highlight the device’s capability to handle short-circuit conditions with minimal voltage fluctuation and a stable gate drive performance up to the point of failure. The extended SCWT, coupled with the controlled tail current, demonstrates the robustness of the 1.7 kV SiC MOSFET under challenging operating conditions. The initiation of degradation at 32.5 µs and eventual failure at 33 µs provide valuable insights for circuit designers, emphasizing the importance of robust gate drive designs to maximize reliability in fault-prone environments.

Under a condition of V_DS_ = 800 V, the SC3 device exhibited thermal runaway as the failure mode, as depicted in Figure 5b. This failure occurred approximately 11 µs after the short-circuit pulse ended, representing a delayed thermal runaway mechanism rather than the conventional failure during the short-circuit pulse. This observation suggests a distinct progression of events where residual thermal and electrical stresses continued to affect the device even after turn-off.

Unlike traditional mechanisms that attribute thermal runaway to immediate parasitic BJT activation or thermal carrier generation, this delayed failure highlights the interplay of localized thermal gradients, material degradation, and time-dependent structural weakening. Post-pulse, the junction temperature had begun to decline, ruling out the peak junction temperature as the sole trigger for the observed collapse of V_DS_ and surge in I_DS_. Residual thermal stress and uneven cooling can create localized hot spots, which contribute to delayed degradation processes. Failures occurring after turn-off could also be attributed to molten aluminum diffusing through the adhesion layer into the P-well/N-drift junction, driven by high die temperatures over the course of several microseconds [13,34].

Moreover, the presence of a tail current (~3.9 A) sustained power dissipation after turn-off, delaying cooling and contributing to thermal feedback that aggravated localized hot spots and intensified material and structural degradation. At 19 µs, the V_DS_ collapses, marking the onset of catastrophic failure. Simultaneously, I_DS_ is increased sharply and the behavior reflects the loss of current control as the device structure degrades progressively. On the other hand, we observe a spike in the gate-source voltage due to residual electric field stress and increased leakage current caused by the time-dependent weakening of the gate dielectric. Thus, residual thermal effects and structural weakening are apparently important failure causes. This shows that high-voltage SiC MOSFET delayed failures are likely due to innovative device designs and efficient thermal management.

### 4.2. Influence of V_GS_

**Figure 6:**
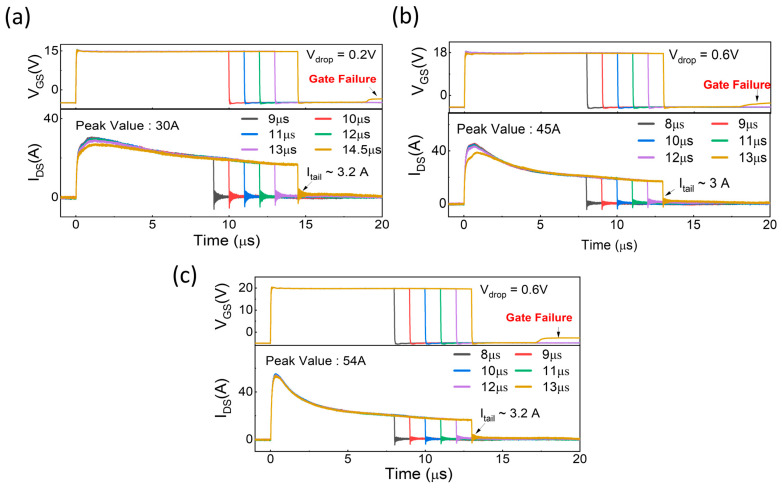
Gate-voltage (V_GS_) and SC current (I_DS_) waveforms of three devices tested at fixed V_DS_ of 600 V with various V_GS._ (**a**). Device SC5 tested under V_GS_ (−5/15 V) (**b**). Device SC6 tested under V_GS_ (−5/18 V) (**c**). Device SC2 tested under V_GS_ (−5/20 V). This figure illustrates the progression of devices from normal operation during SC to the onset of gate failure.

The 1.7 kV SiC MOSFETs were tested at a fixed 600 V with the typical V_GS_ = −5/15 V, −5/18 V, and −5/20 V to assess the device influence under varying gate voltages. The SC waveforms of devices (SC5, SC6, and SC2), including gate-source voltage (V_GS_) and drain current (I_DS_), are shown in Figure 6, presenting the impact of gate drive voltage on SC performance and failure mechanisms.

In Figure 6a, at V_GS_ = −5/15 V, the SCWT was recorded as 14 µs, with an I_tail_ stabilizing at approximately 3.2 A. During the short-circuit event, the gate voltage experienced a minimal drop (V_drop_) of 0.2 V, indicating relatively low stress on the gate oxide. The I_SC, peak_ reached 30 A, reflecting reduced channel conductivity due to the lower gate drive voltage.

Notably, in the V_GS_ waveform, after the device had been turned off for 3.5 µs, V_GS_ exhibited an unexpected rise from −5 V to −3.5 V, suggesting the onset of thermal stress and potential gate-source leakage. Despite this anomaly, the V_DS_ remained stable throughout, indicating effective blocking by the body diode. The failure mechanism was identified as gate dielectric breakdown, marked by electrical changes indicating increased stress on the gate dielectric and the overall integrity of the device.

When tested at V_GS_ = −5/18 V, the SCWT decreased to 12 µs, with I_tail_ stabilizing at 3 A as shown in Figure 6b. The gate voltage exhibited a larger drop (V_drop_) of 0.6 V during the short-circuit event, indicating increased stress on the gate oxide compared to the −5/15 V condition. The I_SC, peak_ reached 45 A, reflecting improved channel conductivity at the higher gate drive voltage. Similarly, in the V_GS_ waveform, after the device has been turned off for 5 µs, V_GS_ unexpectedly rose from −5 V to −3 V, again indicative of thermal stress leading to gate-source leakage. The consistent stability of V_DS_ highlights the continued effectiveness of the body diode. The failure mechanism remained gate dielectric breakdown.

Under V_GS_ = −5/20 V, the SCWT remained at 12 µs, with an I_tail_ of 3.2 A as shown in Figure 6c. The gate voltage drop (V_drop_) is 0.6 V, reflecting the stress on the gate oxide. The I_SC, peak_ reached 54 A, the highest value observed, indicating enhanced channel conductivity due to the increased gate drive voltage. In the V_GS_ waveform, after the device has been turned off for 4.5 µs, V_GS_ rose unexpectedly from −5 V to −2.5 V, a pattern consistent with the other conditions and indicative of thermal stress causing gate-source leakage. Despite irregularities in the V_GS_ waveforms, the drain-source voltage blocking remained intact, whereas the gate-source terminals were found to be shorted upon inspection. Thus, gate dielectric breakdown is once again attributed as the device failure mechanism.

These results show a consistent trend of I_SC, peak_ enhancement and reduction in SCWT with increasing V_GS_ values, along with anomalies in the gate-source voltage waveform. Hence, these observations highlight thermal stress and its impact on the gate, eventually leading to gate failure. These findings underscore the importance of optimizing the gate driver to balance channel conductivity and device resilience for high-voltage applications, especially under high-stress environments.

### 4.3. SC Performance Metrics and Comparison

**Figure 7:**
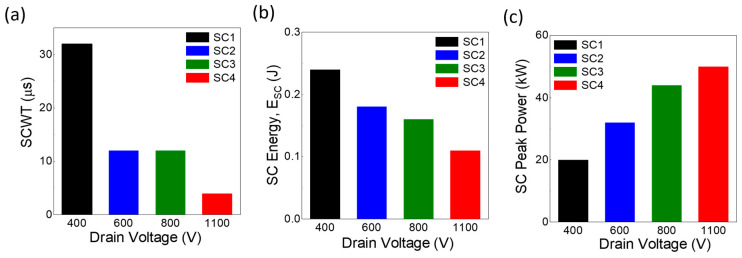
Short-circuit (SC) performance metrics under varying V_DS_ (ranging from 400 V to 1100 V): (**a**) SCWT, (**b**) SC energy, and (**c**) SC peak power.

Figure 7a presents bar chart results for four devices (SC1, SC2, SC3, and SC4) tested under various V_DS_ values of 400 V, 600 V, 800 V, and 1100 V, respectively. The SC1 device exhibited the longest SCWT of 32 µs, followed by SC2 with 12 µs, SC3 with 7 µs, and SC4 with the shortest SCWT of only 4 µs, indicating the worst performance. A clear trend emerges, where SCWT decreases as V_DS_ increases, attributed to enhanced power dissipation and accelerated thermal runaway. This reduction in SCWT with increasing voltage stress highlights a limitation that needs attention to improve device resilience.

Figure 7b illustrates the bar chart results of SC energy (E_SC_) for the same four devices tested under the same V_DS_ values. SC1 achieved the highest E_SC_ of 0.24 J, followed by SC2 with 0.18 J and SC3 with 0.16 J, whereas SC4 exhibited the lowest E_SC_ of 0.11 J. This data reveals a clear downward trend in E_SC_ with increasing V_DS_. This pattern is likely due to enhanced thermal dissipation and reduced energy accumulation time resulting from shorter SCWT at elevated V_DS_. These findings underscore the thermal management capabilities of the 1.7 kV devices.

The SC peak power results, illustrated in Figure 7c, demonstrate a consistent increase with increasing V_DS_. Device SC1 exhibited a peak power of 20 kW at 400 V, SC2 demonstrated 32 kW at 600 V, SC3 attained 44 kW at 800 V, and SC4 achieved 50 kW at 1100 V. These results reflect the higher energy dissipation at elevated voltage conditions while maintaining thermal and structural integrity.

**Figure 8:**
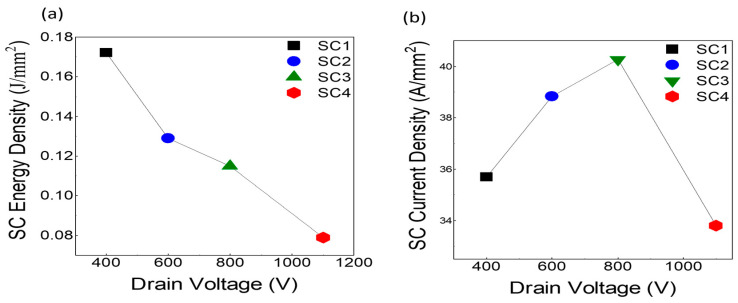
Short-circuit (SC) performance metrics under varying V_DS_ (ranging from 400 V to 1100 V): (**a**) SC energy density and (**b**) SC current density.

SC energy density and SC current density measurements further highlight the robustness of 1.7 kV devices. SC energy density (E_D_) values decreased consistently with increasing V_DS_, with SC1 achieving 0.172 J/mm^2^ at 400 V and SC4 reducing to 0.079 J/mm^2^ at 1100 V, as shown in Figure 8a. Conversely, SC current density demonstrated a peak value of 40.28 A/mm^2^ for SC3 at 800 V before declining to 33.81 A/mm^2^ for SC4 at 1100 V (Figure 8b).

The SC current density increases from 400 V to 800 V due to the enhanced electric field driving higher carrier velocity in the JFET and drift regions, resulting in higher I_SC, peak_**.** However, beyond 800 V, at 1100 V, the SC current density declines could be attributed to the following factors:

- Reduction in peak SC (I_SC, peak_):

Short-circuit current density (JSC) is derived as

J_SC_ = I_SC, peak_/Active Area

- In our experiments, the peak SC current values were measured as 51 A at 400 V, 54 A at 600 V, 56 A at 800 V, and 47 A at 1100 V. This reduction in I_SC, peak_ at 1100 V directly results in a lower SC current density value.
- Thermal effects and phonon scattering: Higher V_DS_ leads to significant power dissipation, causing a temperature rise in the JFET and drift regions. This increases phonon scattering, which reduces carrier mobility and lowers peak SC current, consequently decreasing SC current density.
- Electric field saturation: At 1100 V, the electric field in the JFET region becomes extremely strong, leading to carrier velocity saturation, where further increases in V_DS_ do not proportionally enhance current.
- Localized stress and degradation: High electric field concentrations at extreme V_DS_ cause self-heating and localized degradation in the JFET region, further limiting I_SC, peak_.
- The influence of circuit tolerances on V_GS_: Minor tolerances in the gate drive circuit components could also result in small fluctuations in V_GS_ during the short-circuit event. These fluctuations, while minor, could influence the channel conductivity and therefore impact the SC current. At higher V_DS_ (e.g., 1100 V), these small variations could become more pronounced due to the increased electric field stress, further contributing to the decline in SC current density.

The ability of 1700 V SiC MOSFETs to achieve an SCWT of 12 µs under 600 V test conditions demonstrates their robustness and suitability for high-voltage applications. While 600 V serves as a typical testing condition, it is also relevant for auxiliary power supplies in three-phase converters, where DC bus voltages often fall within a wide range of 300 V to 1000 V [35,36]. By achieving a 12 µs SCWT at 600 V even at elevated V_GS_ levels, these devices represent a significant advancement over 1.2 kV counterparts, which typically achieve only 7 µs under similar electrical stress [27]. These results indicate that 1.7 kV SiC MOSFETs deliver excellent performance and are better suited for applications driven by high performance and reliability, particularly as they surpassed the industry-standard 10 µs SCWT benchmark under 600 V test conditions.

**Figure 9:**
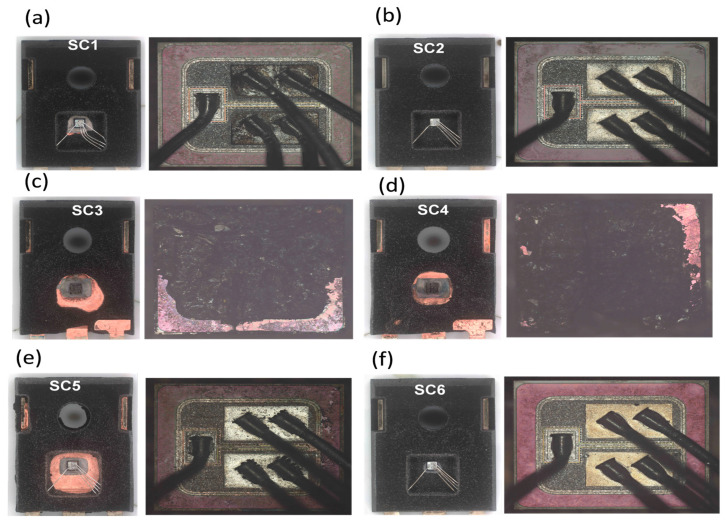
Optical microscope images of decapsulated SiC MOSFETs (SC1–SC6) with enlarged views of chips, after short-circuit testing. (**a**,**b**) SC1 and SC2, tested at 400 V and 600 V, show minimal damage. (**c**,**d**) SC3 and SC4, tested at 800 V and 1100 V, display severe thermal runaway damage with molten metal shorting electrodes. (**e**,**f**) SC5 and SC6, tested at 600 V under varying V_GS_ (−5/16 V, −5/18 V), show intermediate damage. These images illustrate the progressive increase in failure severity with higher voltage stress.

Figure 9 provides optical microscope (OM) images of all tested devices (SC1–SC6) post-decapsulation, highlighting failure mechanisms under various short-circuit (SC) test conditions. Enlarged views of the SiC chips offer detailed inspection of failure points.

Devices SC1 and SC2 (Figure 9a,b), tested at lower voltages (400 V and 600 V), display minimal damage, with SCWTs of 32 µs and 12 µs, respectively. However, SC1 exhibits slightly more damage compared to SC2, likely due to its prolonged SCWT at 400 V. Enlarged views confirm intact wire bonds and minimal material degradation, indicating limited thermal stress.

In contrast, devices SC3 and SC4 (Figure 9c,d), tested at higher voltages (800 V and 1100 V), suffer catastrophic failure. Enlarged views reveal molten metal shorting the electrodes and severe thermal damage across the chip surface, confirming thermal runaway as the dominant failure mechanism. Extensive burn marks and melted structures reflect the significant instantaneous temperature rise during SC events at elevated voltages.

Devices SC5 and SC6 (Figure 9e,f), tested at 600 V under varying V_GS_ conditions (−5/16 V and −5/18 V), exhibit intermediate damage levels, similar to SC2. However, SC5 shows greater physical damage than SC6, attributed to its longer SCWT (14 µs compared to 12 µs).

These results underscore the interplay between SCWT and V_DS_ in determining the extent of thermal and physical damage. While higher V_DS_ accelerates thermal runaway, prolonged SCWT at lower voltages can result in comparable damage. These findings highlight the critical importance of robust thermal and electrical management in device designs for high-power applications.

## 5. Conclusions

In this paper, the short circuit (SC) robustness of commercially available 1.7 kV SiC MOSFETs has been comprehensively evaluated under varying drain-source voltages (400 V, 600 V, 800 V, and 1100 V) and gate-source voltages (−5 V/15 V, −5 V/18 V, and −5 V/20 V). The results showed a clear tradeoff between SC withstand time (SCWT) and applied voltage, with SCWT values decreasing with increasing V_DS_ from 32 µs at 400 V to 4 µs at 1100 V. However, under 600 V—a scenario close to the practical rated voltage for applications—the devices achieved an SCWT of 12 µs, surpassing the conventional industry benchmark of 10 µs. This performance underscores the potential of 1.7 kV SiC MOSFETs for high-voltage applications requiring durability and efficiency.

While no fundamentally new failure mechanisms were observed in the 1.7 kV SiC MOSFET device, the primary failure modes were consistent with those reported for 1.2 kV devices. Moreover, in our study we found that devices tested ≤ 600 V demonstrated gate dielectric or gate degradation failures, while devices tested at 800–1100 V exhibited thermal runaway as the dominant failure mode.

The failure behavior, as characterized electrically and through optical microscopy, suggested signs of gate oxide degradation and thermal runaway, emphasizing the importance of robust gate drive designs and effective thermal management strategies. The influence of gate-source voltage revealed a trade-off between increased channel conductivity and device stability, further reinforcing the need for optimal operating conditions.

This study is limited to the evaluation of SC robustness for a single vendor’s devices. Future studies should extend research to include multiple devices from different vendors and across various generations to enhance the understanding of 1.7 kV SiC MOSFETs. Moreover, the systematic post SC failure should also be analyzed through standard methods, i.e., Lock-in Thermal Emission Microscopy (LITEM) or Focused Ion Beam (FIB) techniques; this could provide deeper insights into physical degradation. The aid of simulations to explore device physics and the detailed analysis on how specific design features affect SC performance is also very important. Exploring these factors will provide deeper insights into SC performance of the emerging 1.7 kV SiC MOSFET and help its widespread adoption in high-power applications.

## Figures and Tables

**Table 1:** Key device parameters mentioned in their datasheets.

Manufacturer	CREE
Structure	Planar
Generation	2G
V_DS_	1700 V
R_DS (on)_	1000 mΩ
I_DS_	5 A
Drive Voltage	−5/20 V
Package	TO-247-3

## Data Availability

The data presented in this study are available on request from the corresponding author.
